# Supplementary material for: Structural basis of adaptor-mediated protein degradation by the tail-specific PDZ-protease Prc
Source: Nat Commun. 2017 Nov 15;8:1516. doi: 10.1038/s41467-017-01697-9 (PMC5686067; doi:10.1038/s41467-017-01697-9)
Supplement: Supplementary file 1 — Supplementary Information [file 41467_2017_1697_MOESM1_ESM.pdf]

SUPPLEMENTARY TABLE

Supplementary Table 1: List of primer sequences used in the study.

sNlpI forward	CATCATCACAGCAGCGGCGAGAATCTCTACTTCCAGGGTCATATGAGTAATACTTCC
sNlpI reverse	GGAAGTATTACTCATATGACCCTGGAAGTAGAGATTCTCGCCGCTGCTGTGATGATG
sNlpI-ΔN forward	TACTTCCAGGGTCATATGACTTTACAGCAGGAAGTG
sNlpI-ΔN reverse	CACTTCCTGCTGTAAAGTCATATGACCCTGGAAGTA
N-6xHis-sMepS forward	CTGACATATGTGTAGTGCAAATAAC
N-6xHis-sMepS reverse	TCAGCTCGAGTTAGCTGCGGCTGAGA
sMepS-6xHis-C forward	GATCCATATGAGTGCAAATAACACCGCA
sMepS-6xHis-C reverse	GATCCTCGAGTTAGTGGTGGTGGTGGTGGTGGCTGCGGCTGAGAACCCG
Prc-noHis forward	CAACCCGCTCCCGTCAAGTAATCTAGAGTCGACCTG
Prc-noHis reverse	CAGGTCGACTCTAGATTACTTGACGGGAGCGGGTTG
Prc-ΔPDZ forward	TGA GTT TGT CGC TGG AAT TCC TCG AAG ACC GCG CGG TTA A
Prc-ΔPDZ reverse	TTA ACC GCG CGG TCT TCG AGG AAT TCC AGC GAC AAA CTC A
Prc-K308W forward	GTGGTTGCCTTAATTTGGGGGCCGAAGGGCAGT
Prc-K308W reverse	ACTGCCCTTCGGCCCCCAAATTAAGGCAACCAC
Prc-L245A forward	ACTGAAATGAGTTTGTCGGCTGAAGGTATTGGCGCAGTG
Prc-L245A reverse	CACTGCGCCAATACCTTCAGCCGACAAACTCATTTCAGT
Prc-L340G forward	CGTGAACGTATTCGTGGCGAAGACCGCGCGGTT
Prc-L340G reverse	AACCGCGCGGTCTTCGCCACGAATACGTTCACG
Prc-L340A forward	CGTGAACGTATTCGTGCTGAAGACCGCGCGGTT
Prc-L340A forward	AACCGCGCGGTCTTCAGCACGAATACGTTCACG
sNlpI-D113A forward	CAGGCAGGCAATTTTGCGGCTGCCTATGAAGCG
sNlpI-D113A reverse	CGCTTCATAGGCAGCCGCAAAATTGCCTGCCTG
sNlpI-E117A forward	TTTGATGCTGCCTATGCGGCGTTTGATTCTGTA
sNlpI-E117A reverse	TACAGAATCAAACGCCGCATAGGCAGCATCAAA
sNlpI-D120A forward	GCCTATGAAGCGTTTGCGTCTGTACTTGAGCTT
sNlpI-D120A reverse	AAGCTCAAGTACAGACGCAAACGCTTCATAGGC
sNlpI-E124A forward	TTTGATTCTGTACTTGCGCTTGATCCAACTTAC
sNlpI-E124A reverse	GTAAGTTGGATCAAGCGCAAGTACAGAATCAAA
sNlpI-D113A/E117A forward	CAGGCAGGCAATTTTGCGGCTGCCTATGCGGCGTTTGATTCTGTA
sNlpI-D113A/E117A reverse	TACAGAATCAAACGCCGCATAGGCAGCCGCAAAATTGCCTGCCTG
sNlpI-QM forward	GCCTATGCGGCGTTTGCGTCTGTACTTGCGCTTGATCCAACTTAC
sNlpI-QM reverse	GTAAGTTGGATCAAGCGCAAGTACAGACGCAAACGCCGCATAGGC
sNlpI-L38A forward	GTACCATTGCAACCGACTGCACAGCAGGAAGTGATTCTG

sNlpI-L38A reverse	CAGAATCACTTCCTGCTGTGCAGTCGGTTGCAATGGTAC
sNlpI-L38C forward	ACCATTGCAACCGACTTGCCAGCAGGAAGTGATTC
sNlpI-L38C reverse	GAATCACTTCCTGCTGGCAAGTCGGTTGCAATGGT
sNlpI-R82E forward	GGTCTGAGGGCATTAGCGGAAAACGATTTTTCGCAAGCG
sNlpI-R82E reverse	CGCTTGCGAAAAATCGTTTTCCGCTAATGCCCTCAGACC
sNlpI-A114W forward	GCAGGCAATTTTGATTGGGCCTATGAAGCGTTT
sNlpI-A114W reverse	AAACGCTTCATAGGCCCAATCAAAATTGCCTGC
sNlpI-A114C forward	GCAGGCAATTTTGATTGTGCCTATGAAGCGTTT
sNlpI-A114C reverse	AAACGCTTCATAGGCACAATCAAAATTGCCTGC

SUPPLEMENTARY FIGURES

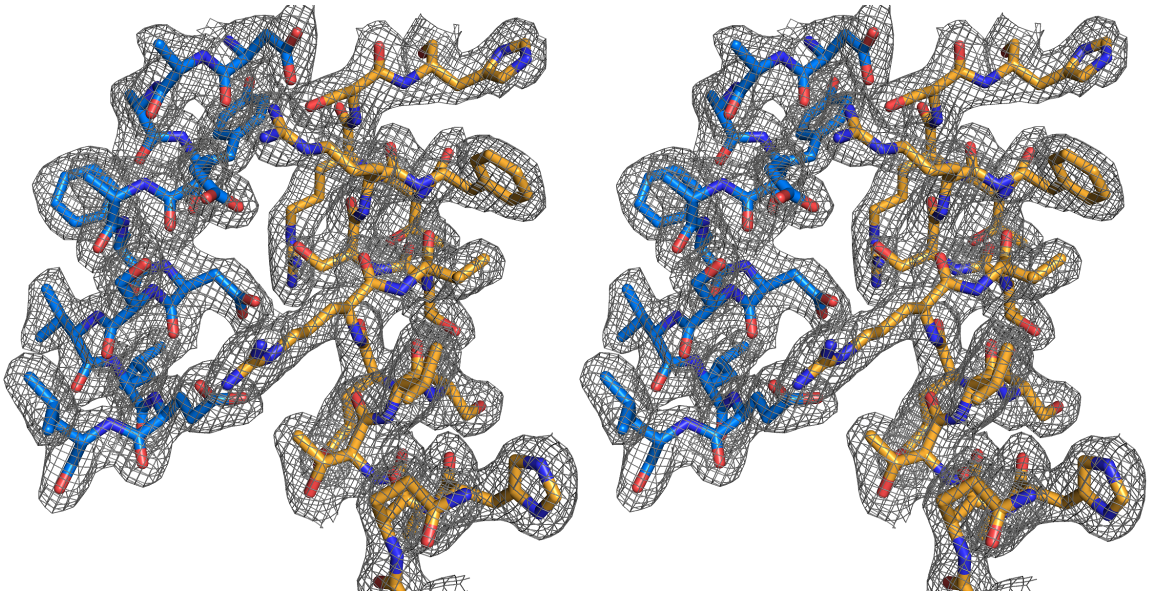

Supplementary Figure 1: Stereo image of the electron density map of the binding interface between NlpI and Prc. 2Fo-Fc electron density map contoured at 1.0 σ level is shown around helix TPR2b (blue) of NlpI and helix h1 (orange) of Prc at the binding interface.

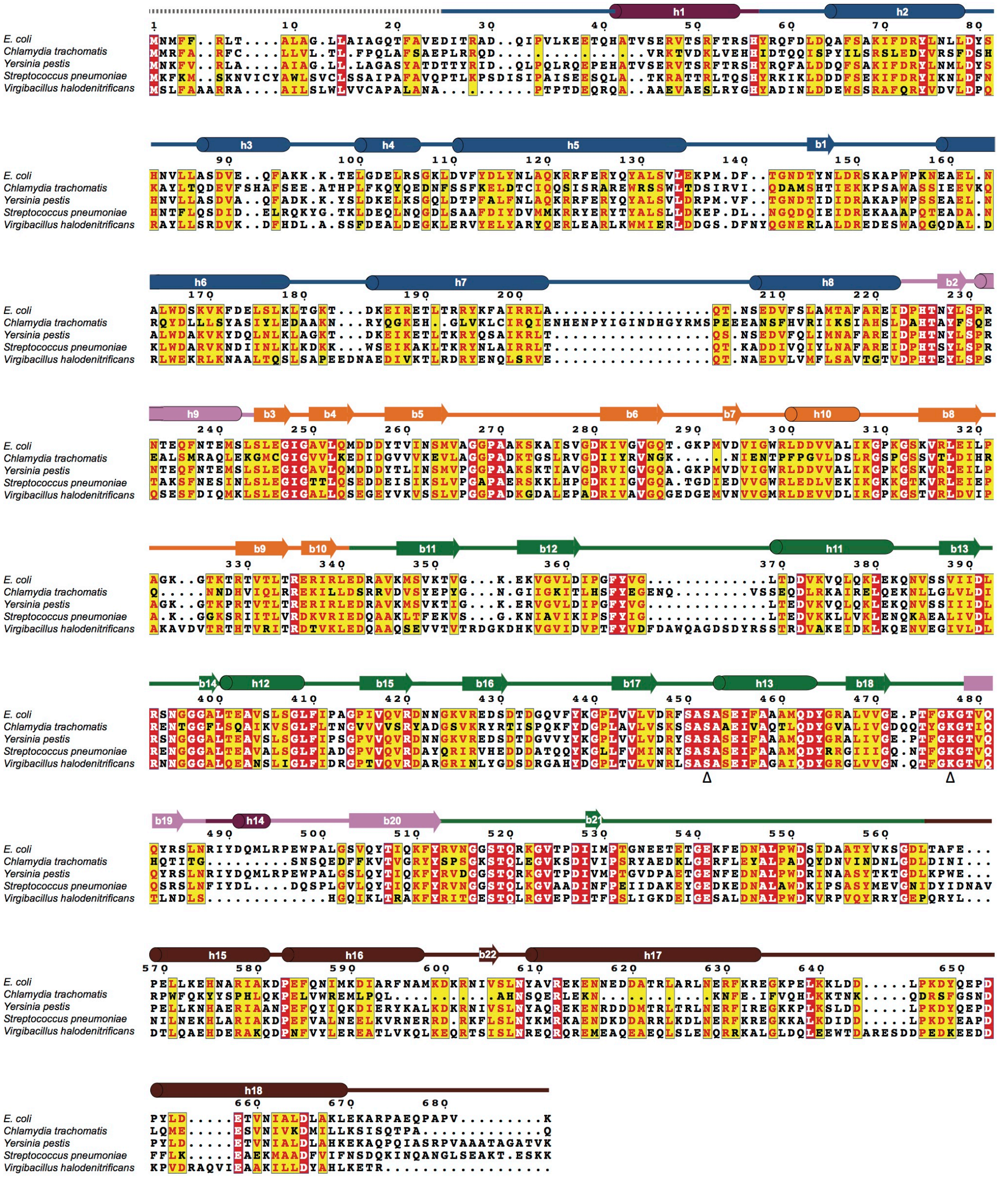

Supplementary Figure 2: Structure-based multiple sequence alignment of bacterial Prc proteases. Strictly conserved residues are colored in white and highlighted with red background. Conservatively substituted residues are boxed and colored in red with yellow background. The catalytic residues are indicated by open triangles. The secondary structural elements (α helices: h; β strands: b) are shown above the aligned sequences and colored in the same scheme as in Fig. 3. Sequences used include *E. coli* Prc (NCBI accession number: NP_416344.1) and homologues from Gram-negative bacteria including *C. trachomatis* (WP_009873811.1), *Y. pestis* (WP_002210848.1) and Gram-positive bacteria including *S. pneumoniae* (CVP79417.1), and *V. halodenitrificans* (CDQ33021.1). The alignment was performed with Expresso and displayed using ESPript 3 [1,2].

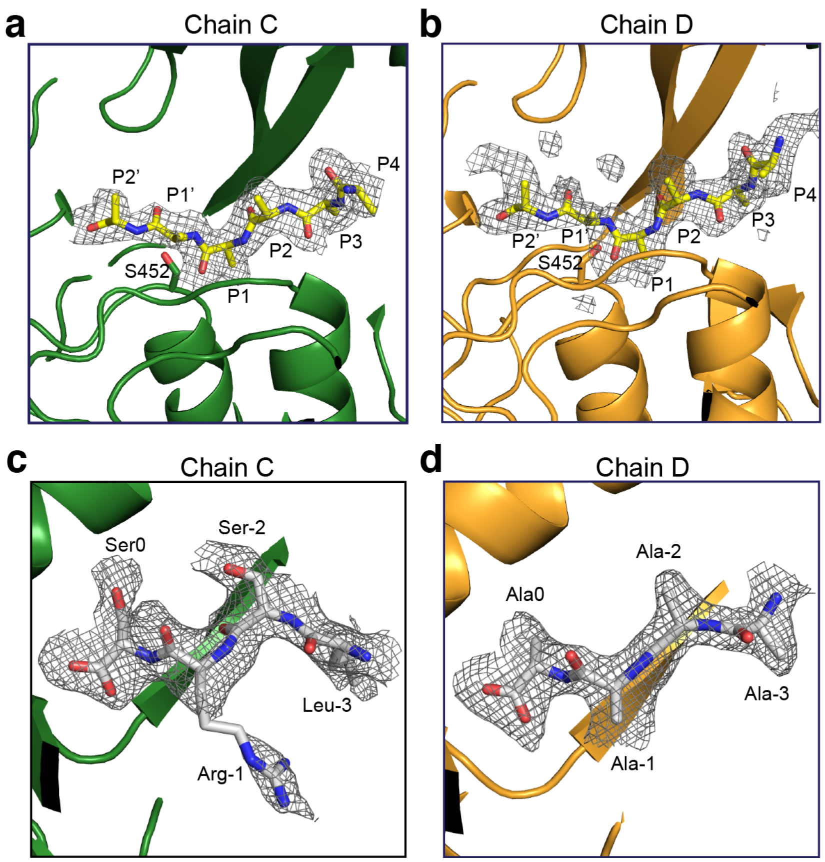

Supplementary Figure 3: Co-crystallized peptides bound to Prc. (a-b) Fo-Fc omit map of co-purified peptides bound to the substrate-binding passage, at the proteolytic site in chain C (a) and chain D (b). The catalytic residue S452 is labeled. The peptides were modeled as poly-Ala. The contour level of the map is 2.0 σ. (c) Fo-Fc omit electron-density map of the co-purified peptide bound to the PDZ domain in chain C. The peptide was tentatively modeled as LSRS-COOH. The contour level of the map is 1.8 σ. (d) Fo-Fc omit map of the co-purified peptide bound to the PDZ domain in chain D. The peptide was tentatively modeled as AAAA-COOH. The contour level of the map is 2.0 σ.

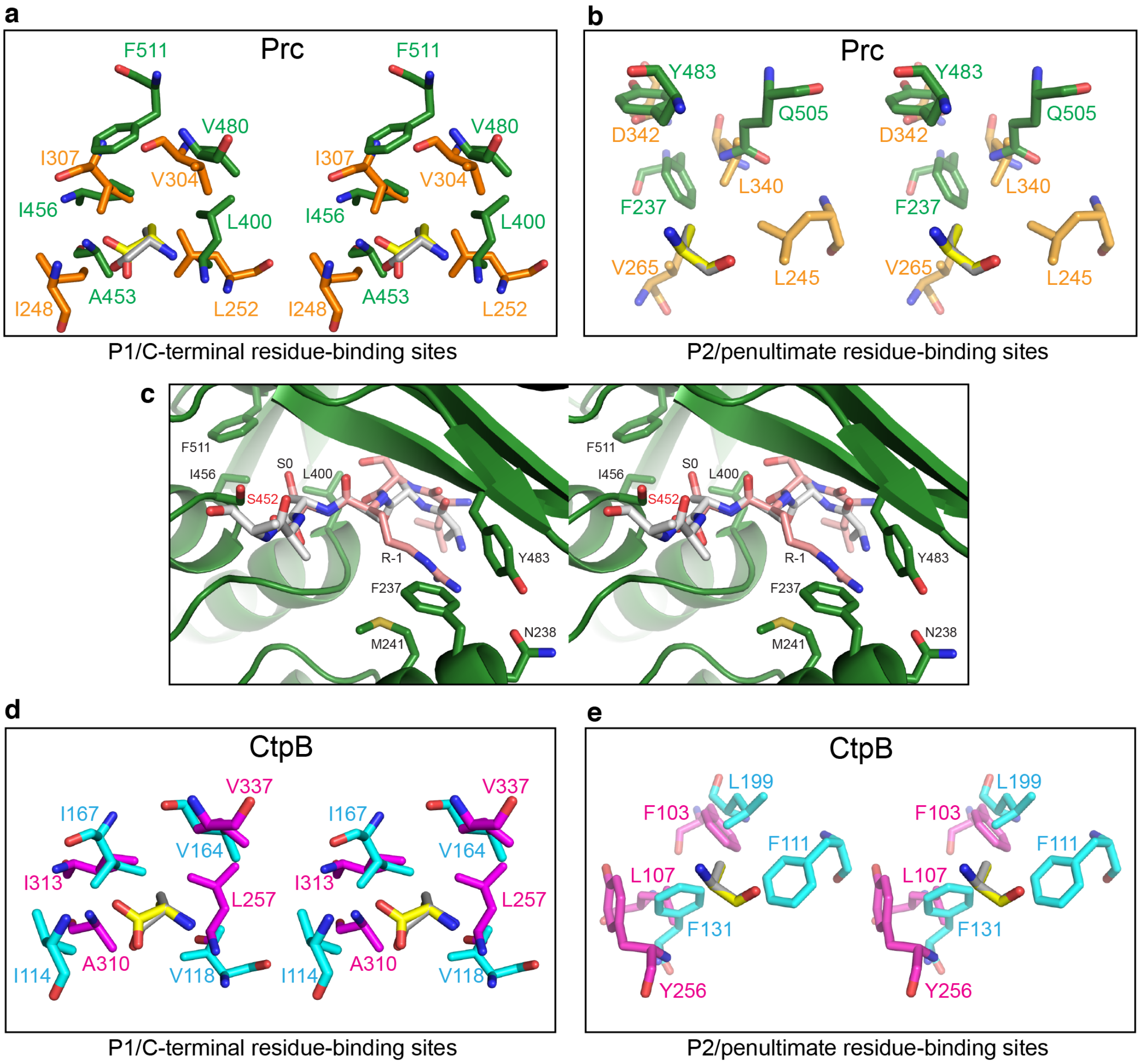

Supplementary Figure 4: Structural comparison of substrate *versus* PDZ-ligand binding pockets in Prc and CtpB. (a and b) Structures superimposed of the P1 (grey) with the C-terminal (yellow) residues (a), and of the P2 (grey) and the penultimate (yellow) residues (b) of the peptides bound to the proteolytic active site and the PDZ domain, respectively, of Prc. (c) Structure of the PDZ-binding peptide LSRS-COOH docked to the proteolytic site by superposition with the P1-P2 residues of the bound poly-Ala peptide fragment in Prc (chain C), yielding an r.m.s.d. of 0.34 Å. The catalytic residue S452 is labeled in red. (d and e) Superimposed P1/C-terminal residue binding sites (d) and P2/penultimate residue binding sites (e) in CtpB. The substrate/ligand residues are represented by alanines in sticks for clarity. Residues of the proteolytic and PDZ ligand-binding sites are shown in green and orange sticks, respectively, in Prc (chain C) and in magenta and cyan sticks, respectively, in CtpB.

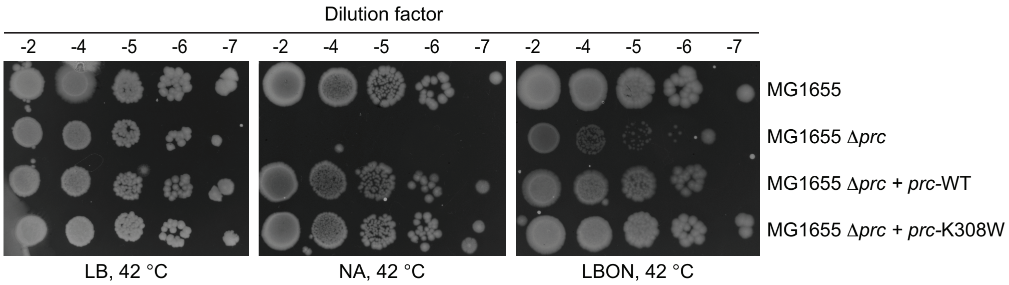

Supplementary Figure 5: **Effect of expressing wild-type Prc and Prc-K308W in Δ*prc* mutant cells.** Cultures of WT MG1655 or MG1655Δ*prc* cells carrying the *prc*-WT or *prc*-K308W plasmids were grown in LB broth with ampicillin and 5 µl of various dilutions were spotted on indicated plates and grown overnight.

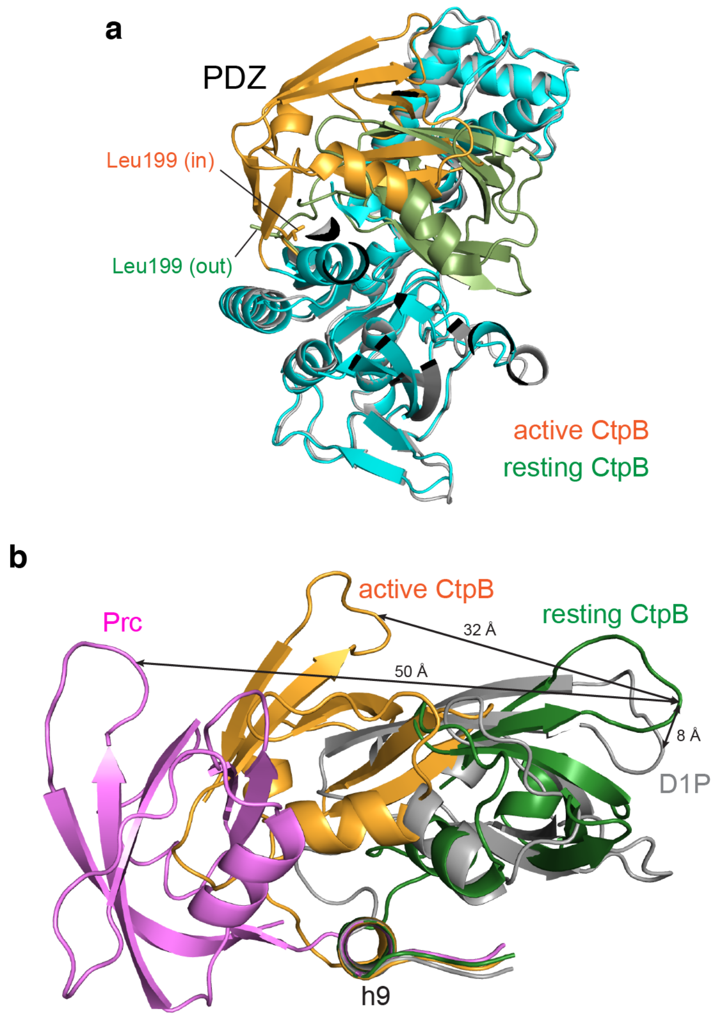

Supplementary Figure 6: **Structural analysis of the PDZ domain in open and closed states.** (a) Cut-away view perpendicular to the substrate passage of the active and resting CtpB structures superimposed based on the protease core showing the distinct "in" and "out" locations, respectively, of L199 (PDB codes 4C2C and 4C2E). The PDZ domains in the resting and active CtpB are colored in green and gold, respectively. (b) Structural superposition based on helix h9 showing the locations of the linked PDZ domains in Prc (chain C; this work), active CtpB, resting CtpB, and D1P (PDB code 1FC7). The distances are measured between the conserved glycine residues in the loops of the homologous PDZ domains.

SUPPLEMENTARY REFERENCES

1. Armougom, F. et al. Expresso: automatic incorporation of structural information in multiple sequence alignments using 3D-Coffee. *Nucleic Acids Res* **34**, W604-8 (2006).
2. Robert, X. & Gouet, P. Deciphering key features in protein structures with the new ENDscript server. *Nucleic Acids Res* **42**, W320-4 (2014).
